# Case Report: Isolated Radial Collateral Ligament Thumb Tear in a Teenage Cheerleader Base: A Rare Injury from an Overhead Stunt

**DOI:** 10.5811/cpcem.50606

**Published:** 2026-03-29

**Authors:** Russell Baker

**Affiliations:** Texas Tech University Health Sciences Center, Department of Emergency Medicine, El Paso, Texas

**Keywords:** thumb injury, radial collateral ligament, cheerleading, adolescent, stunting, case report

## Abstract

**Introduction:**

Radial collateral ligament injuries of the thumb are rare, especially in adolescent athletes. We present a case of a 17-year-old female cheerleader who sustained a complete radial collateral ligament tear while basing during a cheerleading stunt.

**Case Report:**

The patient presented to the emergency department with pain in the right thumb after catching a falling flyer. Examination of the first metacarpophalangeal joint of the right thumb revealed tenderness and laxity. Radiographic imaging showed no fracture. Magnetic resonance imaging confirmed a complete radial collateral ligament tear. She underwent surgical repair with full recovery.

**Conclusion:**

This case highlights an uncommon thumb ligament injury in a non-traditional mechanism. Emergency physicians should consider radial collateral ligament tears in patients with metacarpophalangeal joint tenderness, even when radiographs are normal.

## INTRODUCTION

Collateral ligament injuries of the thumb metacarpophalangeal joint are common in sports involving falls, torsional forces, or high-load gripping. The ulnar collateral ligament is involved in most of these injuries, typically due to valgus stress, and is classically referred to as “skier’s thumb” or “gamekeeper’s thumb.”^1^ Radial collateral ligament injuries are far less common, accounting for a small minority of cases, and are rarely reported in pediatric or adolescent athletes.^2^ Cheerleading is now recognized as a high-demand athletic activity with over 3.5 million participants in the United States.^3^ While injury surveillance data consistently identify high rates of musculoskeletal trauma, particularly to the upper extremities during stunting, radial collateral ligament injuries have not been specifically associated with cheerleading. This case describes an isolated, complete radial collateral ligament tear sustained by a base during a complex aerial stunt, emphasizing the diagnostic considerations and implications for emergency physicians.

## CASE REPORT

**Image 1:**
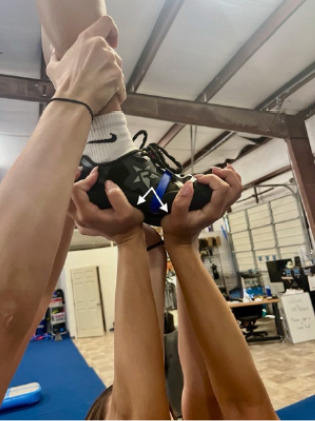
Hand position of cheerleading base for a two-leg extension. White arrows indicate the thumb metacarpophalangeal joint and the potential area for sudden adduction force.

A 17-year-old, right hand-dominant female competitive cheerleader presented to the emergency department (ED) with right thumb pain following a stunting injury. She was acting as a base during a “full twisting rewind” stunt. During the catch, the flyer descended off axis, and the patient’s right thumb was forced into abrupt hyperextension and radial deviation as she caught the flyer’s foot. Image 1 demonstrates proper catch and hand placement.

On physical examination of the thumb, there was localized tenderness over the radial aspect of the metacarpophalangeal joint, minimal swelling, and no ecchymosis. Varus stress testing revealed increased laxity compared to the contralateral thumb, with a soft endpoint. Sensation to light touch and two-point discrimination was intact in both the radial and ulnar digital nerve distributions, and capillary refill was normal. No associated wrist or scaphoid tenderness was present. Plain radiographs of the right thumb, including stress views, revealed no fracture, subluxation, or joint space widening. The patient was immobilized in a thumb spica splint and referred for outpatient follow-up.

**Image 2:**
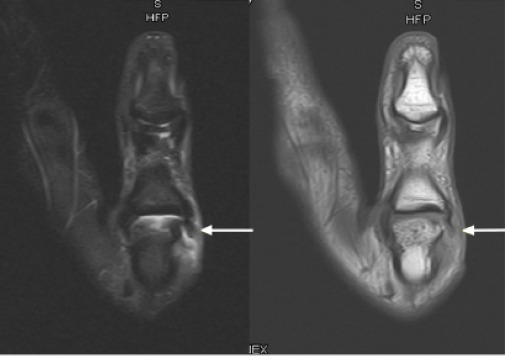
T1- (left) and T2- (right) weighted magnetic resonance imaging of the right thumb metacarpophalangeal joint demonstrating a complete tear of the proximal radial collateral ligament (white arrows).

At one-month follow-up with orthopedics, the patient reported persistent pain and instability. In week five, 3.0 Tesla magnetic resonance imaging was recommended and demonstrated a complete tear of the radial collateral ligament at its metacarpal origin, along with mild dorsal subluxation of the proximal phalanx (Image 2). The patient underwent surgical repair within two weeks of confirmatory diagnosis, consisting of reattachment of the ligament to the metacarpal head using a Mitek suture anchor. Postoperatively, she completed a period of immobilization followed by supervised hand therapy. At 12 weeks, she demonstrated full range of motion, grip strength equal to the contralateral side, and return to non-contact cheerleading.

## DISCUSSION

The radial collateral ligament of the thumb metacarpophalangeal joint functions as the primary restraint to varus stress and contributes to joint stability during pinch and grip.^4^ While ulnar collateral ligament injuries are common due to abduction and valgus loading, radial collateral ligament injuries occur less frequently and are often overlooked during initial evaluation. Failure to recognize these injuries can result in chronic metacarpophalangeal instability, decreased pinch strength, and early degenerative arthropathy.^5^

*CPC-EM Capsule*

What do we already know about this clinical entity?

*Injuries to the thumb radial collateral ligament are uncommon and frequently overlooked in initial assessment*.

What makes this presentation of disease reportable?

*A complete radial collateral ligament tear occurred in a teenage cheerleading base, representing a novel sports-related injury mechanism*.

What is the major learning point?

*Radial collateral ligament injury should be suspected in patients with metacarpophalangeal tenderness and normal imaging after high-energy trauma*.

How might this improve emergency medicine practice?

*The case promotes early recognition and referral of thumb instability to prevent chronic pain and functional loss*.

This case represents an uncommon mechanism of radial collateral ligament disruption in a cheerleading base. Injury biomechanics involved a high-energy varus load transmitted axially through the thumb as the athlete attempted to control the flyer’s fall. Epidemiologic data from the National Electronic Injury Surveillance System and cheerleading injury registries demonstrate that stunting is the leading cause of upper extremity injury, accounting for approximately 29% of reported cases, but injury categorization rarely differentiates between collateral ligament types.^6^ This suggests that radial collateral ligament injuries may be under-reported or misclassified.

From an emergency medicine standpoint, evaluation of thumb trauma should include bilateral metacarpophalangeal joint stress testing to assess varus and valgus stability. The exam should document neurovascular status and compare findings to the uninjured hand. Targeted radiographs, including stress views if tolerated, are useful to exclude associated avulsion fractures or subluxations. If significant instability is present despite normal radiographs, early referral for advanced imaging is recommended. Prompt identification is crucial, as surgical repair is typically indicated for complete radial collateral ligament ruptures, especially when joint subluxation or persistent instability is present.^7^ Delayed diagnosis is associated with inferior outcomes, including chronic pain and reduced grip strength.^8^

## CONCLUSION

Isolated radial collateral ligament tears of the thumb metacarpophalangeal joint are rare and may be missed during the initial ED evaluation, particularly in sports not traditionally associated with this injury pattern. This case highlights an increasingly prevalent mechanism in cheerleading and underscores the importance of thorough ligamentous testing in any patient with metacarpophalangeal joint tenderness following high-energy upper extremity trauma. Early recognition, appropriate immobilization, and timely referral can optimize functional outcomes.^9^

## References

[1] Patel DS, Lipman GS, Monto RR (2011). Thumb injuries in athletes. Curr Sports Med Rep.

[2] Mitchell TW, deSa DL (2022). Radial collateral ligament injuries of the thumb MCP joint: epidemiology, biomechanics, diagnosis, and treatment. Hand (N Y).

[3] American Academy of Pediatrics, Council on Sports Medicine and Fitness (2012). Cheerleading injuries: epidemiology and recommendations for prevention. Pediatrics.

[4] Avery DM, Rodner CM (2017). Thumb collateral ligament injuries in the athlete. Curr Sports Med Rep.

[5] Shields BJ, Smith GA (2009). Cheerleading-related injuries in the United States: a prospective surveillance study. J Athl Train.

[6] Canty G, King J, Council on Sports Medicine and Fitness (2024). Safety in cheerleading: epidemiology and recommendations: policy statement. Pediatrics.

[7] Sochol KM, Charen DA, Kim J (2018). Upper extremity injuries in pediatric athletes: trends in soft tissue injury. Ann Joint.

[8] American Medical Society for Sports Medicine (2021). AMSSM position statement: cheerleading injuries and prevention. Clin J Sport Med.

[9] Xu AL, Suresh KV, Lee RJ (2021). Progress in cheerleading safety: update on the epidemiology of cheerleading injuries presenting to US emergency departments, 2010–2019. Orthop J Sports Med.

